# Preliminary study for the application of Raman spectroscopy for the identification of Leishmania infected dogs

**DOI:** 10.1038/s41598-022-11525-w

**Published:** 2022-05-06

**Authors:** Acri Giuseppe, Falcone Annastella, Claudia Giannetto, Giudice Elisabetta, Piccione Giuseppe, Testagrossa Barbara, Luca Cicero, Giovanni Cassata, Di Pietro Simona

**Affiliations:** 1 Department of BIOMORF, University of Messina, Via Consolare Valeria, 98125 Messina, Italy; 2 Department of Veterinary Sciences, Polo Universitario Dell’Annunziata, University of Messina, 98168 Messina, Italy; 3 Experimental Zooprophylactic Institute of Sicily “A. Mirri”, Palermo, Italy

**Keywords:** Biological techniques, Infectious diseases

## Abstract

Raman spectroscopy is a rapid qualitative and quantitative technique that allows the simultaneous determination of several components in biological fluids. This methodology concerns an alternative technique to distinguish between non-healthy and healthy subjects. Leishmaniasis is a zoonosis of world interest, the most important agent is L. infantum. Dogs are the principal reservoirs affected by a broad spectrum of clinical features. During a clinical exam, blood samples were collected in tubes without anticoagulants, from twenty two dogs. One aliquot was used for serological test for Leishmaniasis, one aliquot was subjected to the Raman spectroscopic analysis. Animals were divided into two groups of equal subjects, Leishmania group (LG) constituted by infected dogs, and control group (CG) constituted by healthy dogs. The acquired spectra were different in the region 1200–1370 cm^−1^, in which it is possible to distinguish the amide III vibration (~ 1300 cm^−1^). In LG, an evident shift to the shortwave region is observed in spectral frequencies of the band centered at ~ 1250 cm^−1^. Our results distinguished between LD group and CG. Further studies are necessary to exclude the effect of metabolic modification due to disease on the recorded spectra changes and to consolidate the achievability of Raman spectroscopy as rapid and less expensive diagnosis of Leishmaniasis.

## Introduction

Leishmaniasis is a zoonosis caused by parasites of the genus Leishmania, and so it has become a priority in public health all over the world and it is considered by the WHO (World Health Organization) as one of the most important diseases for its lethality as well as its dispersion in several continents with large population groups under infection risk^1^. The leishmanias are protozoan parasites transmitted to a susceptible host by sandflies of the genera Phlebotomus in the Old World and Lutzomyia in the New World^2^. Some studies claim that Leishmania spp. can also be transmitted by arthropods, such as ticks and fleas^3^. There are at least 12 species of Leishmania, some of these are L. infantum, L. tropica, L. major, L. donovani, L. braziliensis, and L. mexicana. The most important etiological agent is L. infantum, widespread in South America and in the Mediterranean region^4^. Domestic animals, especially dogs, represent reservoirs of human visceral leishmaniasis caused by L. infantum (WHO 1990), whereas humans are an occasional host^5^. There are several predisposing factors for the development of disease including breed, age (younger than 3 years and older than 8 years), and genetic background. Some dog breeds such as the Boxer, Cocker Spaniel, Rottweiler, and German Shepherd seem to be more susceptible to the development of disease, while others such as the Ibizian Hound rarely develop clinical signs. The broad spectrum of clinical features depends on the phase of the disease, the state of immunity and, eventually, the previous therapy. Classical canine leishmaniasis appears as a chronic wasting disease with pale mucous due to anemia, generalized or symmetrical lymphadenomegaly, intermittent pyrexia and cutaneous lesions with dry exfoliative dermatitis, ulcerations and alopecia. In more advanced forms there may be eye symptoms, several renal failure, splenomegaly, weight loss, onycogryphosis, anorexia or dysorexia, nodules, ascites, epistaxis. The main clinical signs reported were weakness, skin problems, anorexia and weight loss^6^. Diagnosis is very difficult because the disease often manifests itself in an atypical form or with few or non-specific clinical symptoms. Another way to consider is the presence of other infections such as canine ehrlichiosis or rickettsiosis, which produce clinical signs similar to leishmaniasis and therefore do not allow a correct diagnosis and after a targeted therapeutic approach^6^. The basic way is characterized by the observation of clinic symptoms, laboratory analyzes with complete blood count (CBC), biochemical profile, urinalysis and cytology or histology, parasitological or serological tests, ELISA, and others. Laboratory analyzes can be altered; the infected dogs can develop moderate normochromic normocytic anemia; white blood cells initially were slightly higher than normal with an absolute neutrophilia. They can develop lymphocytosis in association with both normal and low total cell white counts. In the infected dogs, clinical chemistry and urinalysis may indicate renal dysfunction (azotemia, decreased urine specific gravity, proteinuria) and an inflammatory/immune response total protein and globulin values become moderately elevated (acute phase proteins APP or alfa2 and/or gamma-globulins), and albumin was decreased^7^. The diagnosis of leishmaniasis can be made by detection of specific serum antibodies (IgG) using preferably quantitative serological techniques, such as the immunofluorescence antibody test (IFAT) and enzyme-linked immunosorbent assay (ELISA) and a classification of the level of antibodies. Detection of Leishmania DNA in tissues by PCR allows sensitive and specific diagnosis of infection and it allows quantification of the Leishmania parasite load in the tissues of infected dogs, which is useful for the diagnosis and the follow-up during treatment^8^.

In this context, Raman spectroscopy could represent a valid methodology for the screening of Leishmania diffusion in symptomatic and asymptomatic dog populations in view of its diagnostic application. Raman spectroscopy is an inelastic light-scattering phenomenon according to which the illumination of a molecule by a monochromatic laser beam will give rise to an exchange of a quantum vibrational energy between the two, which will result in a difference in vibrational frequency between incident and scattered light^9,10^. Consequently, this experimental method provides a vibrational spectrum that contains information relative to chemical bonds and symmetry of a specific molecule^11^. It represents an essential methodology in chemistry, physics, biology, material science and medicine^12–14^. Multidisciplinar research has shown that Raman spectroscopy combined with advanced statistics can determine differences in molecular structure and biochemical composition of body fluids and tissues^15–17^. Moreover, Raman spectroscopy has proved to be a versatile tool in clinicam diagnostics^18^, applied on tissues in order to detect a variety of diseases ranging from cancer^18–20^ to infectious diseases^21^, neurodegenerative diseases^22^ and inflammatory diseases^23–25^.

The aim of this preliminary study concerns an alternative methodology for the screening of Leishmania, in order to simplify procedures and provide a versatile and fast method to distinguish between infected and uninfected animals. The reliability of the proposed alternative methodology was evaluated by conducting statistical analysis. In particular, we used the receiver operating characteristic (ROC) curve and the Youden index (Y), with its associated cut-off point in order to distinguish infected dogs from healthy ones.

## Materials and methods

### Animals

Twenty-two dogs subjected to a clinical exam at the University Veterinary Teaching Hospital (OVUD) of Messina University were enrolled in the study. Dogs of various breeds and sex (Table 1) were divided into two groups with the same number of animals on the basis of the results of their clinical exam, hematological and hematochemical analyses and serological test for leishmaniasis performed during the clinical examination. Diagnosis of leishmaniasis was established based on the indications reported in the Canine Leishmaniasis Working Group (CLWG)^26,27^.

**Table 1:** Anamnesis of the twenty-two dogs enrolled in the study.

Subject	Breed	Gender	Age (years)	Cut-off 1:80
**Leishmaniosis group**				
LG 1	Pitt bull terrier	♂	2	1:320
LG 2	Mixed breed	♀	4	1:1280
LG 3	Mixed breed	♂	1	1:10240
LG 4	Mixed breed	♀	5	1:1280
LG 5	German Shepherd	♀	6	1:1280
LG 6	Mixed breed	♀	9	1:320
LG 7	Dogo argentino	♂	9	1:1280
LG 8	Mixed breed	♀	5	1:320
LG 9	Rottweiler	♂	5	1:160
LG 10	Mixed breed	♀	3	1:5120
LG 11	Malamute husky	♀	3	1:320
**Control group**				
CG 1	Pug	♀	4	
CG 2	Pug	♂	5	
CG 3	Labrador retriver	♂	3	
CG 4	Jack russel	♀	5	
CG 5	Labrador retriver	♂	2	
CG 6	Mixed breed	♀	2	
CG 7	Mixed breed	♀	6	
CG 8	Mixed breed	♂	6	
CG 9	Rottweiler	♀	4	
CG 10	Mixed breed	♂	7	
CG 11	Mixed breed	♂	1	

Leishmania group (LG) was constituted by 11 dogs showing clinical signs referred to the infectious disease and classified as being in an “ILL” C stage, meaning infected dogs in which was possible to demonstrate the presence of the parasite or antibody titers (IFAT) over 4 times the threshold value of the reference laboratory (Experimental Zooprophylactic Institute of Sicily; cut-off 1:80). The control group (CG) was constituted by 11 clinically healthy dogs.

Blood samples collected during the clinical exams in tubes without anticoagulant were centrifuged at 1.785*g* for 10 min and stored into two aliquots, one for the assessment of the hematochemical parameters linked to the clinical exam, and one was frozen at − 20 °C and stored until the Raman spectroscopic analysis performed at Department of Biomedical, Dental, Morphological and Functional Imaging Sciences of Messina University.

### Ethics declaration

All treatments and animal care reported previously were carried out following the standards recommended by the European Directive 2010/63/EU for animal experiments. Ethical approval from a committee was therefore not required. All animals were en-rolled in the study after the written consent of the owners in compliance with the Italian Regulation D.L. 116/1992.

### FT Raman spectroscopy

All the Raman spectroscopy measurements were performed by means a DXR-SmaryRaman Spectrometer (Thermo Fisher Scientific) using a diode laser with an excitation wavelength of 785 nm. All the Raman spectra were acquired over the wavenumber range of 3300–400 cm^−1^, with a resolution of 1.9285 cm^−1^ and irradiated with a laser power of 24 mW, coming out from 50 μm spot (estimated spot size on the sample 3.1 μm).

Vials containing serum were accommodated into their sample holder and the 180 Degree Sampling Accessory was used for measurements. In order to obtain high signal-to-noise ratio (S/R) spectra, each Raman spectrum was obtained after collecting 32 sample exposures, and the duration of each exposure during data collection was set equal to 30.0 s. The total acquisition time was 16 min for each spectrum. To obtain adequate information from acquired spectra, we performed a manual baseline correction of each of them, picking points that define the new baseline. This baseline is subtracted from the spectrum to yield the baseline corrected spectrum. The last step is represented by the normalization of each spectrum to the total integrated area.

**Figure 1:**
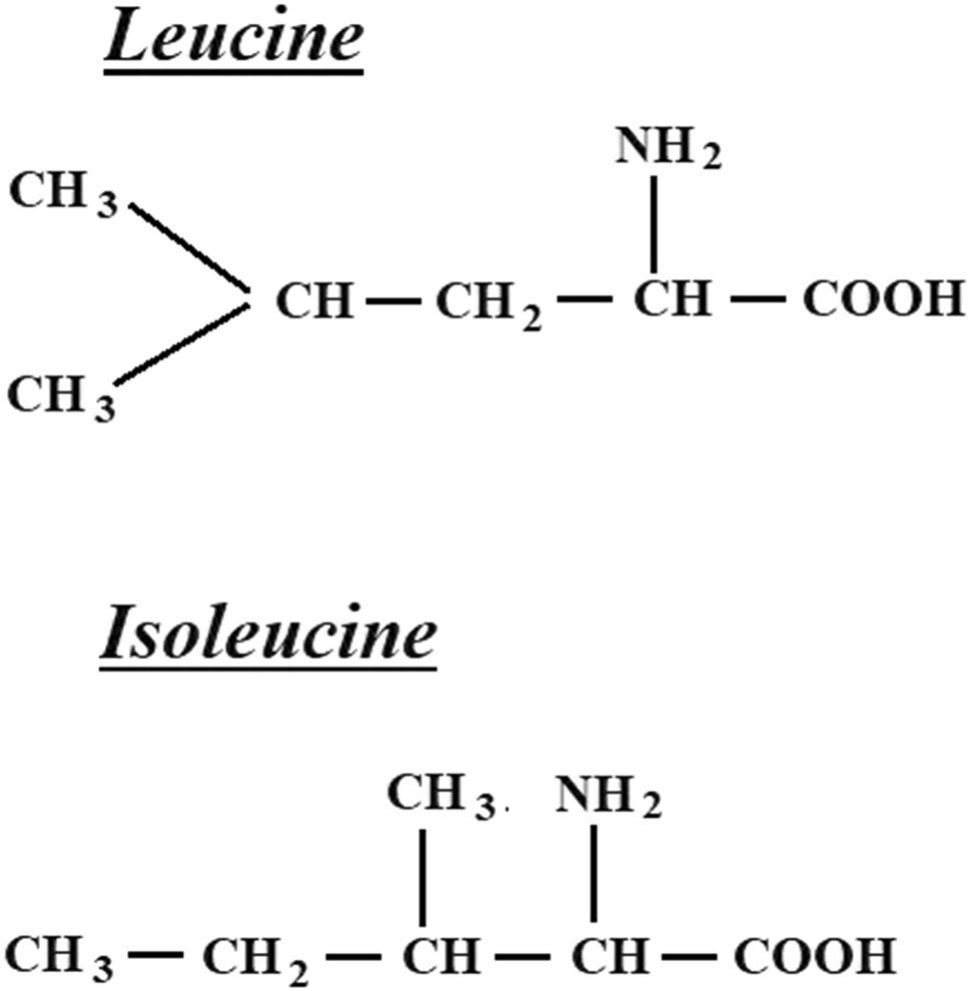
Structural formula of leucine and isoleucine.

From obtained spectra, the region 1200–1370 cm^−1^, corresponding to the Amide III^28^, was considered. Inside it, we took into account two spectral ranges for evaluation: the 1213–1279 cm^−1^ spectral range and the 1280–1369 cm^−1^ one. The 1213–1279 cm^−1^ region corresponds to the CH2 torsion in leucine and isoleucine^29,30^ and C–O–H deformation in leucine^31^; the 1280–1370 cm^−1^ band is assigned to the CH deformation in leucine^32^. For each acquired spectrum the baseline correction and the normalization to the total integrated area were performed. The overall area of the above two spectral ranges used for diagnosis was obtained using the “integrated” function available on the Origin software. The spectral bands were analyzed by a curve-fitting procedure in order to evaluate the overall area of each band and hereafter were indicated using their frequency centers, as A1350 and A1250 and the A1350/A1250 ratio has been computed. Figure 1 shows the structural formula of leucine and isoleucine.

### Statistical analysis

The results of many clinical tests are quantitative and are provided on a continuous scale. To help decide the presence or absence of disease, a cut-off point must be chosen. The receiver operating characteristic (ROC) curve is used to evaluate a biomarker’s ability for classifying disease status. A ROC curve represents the plot of sensitivity vs 1-specificity^33^. In this study, the sensitivity referred to the ability of Raman spectroscopy to correctly identify those subjects with the LD; instead, the specificity referred to the ability of Raman spectroscopy to correctly identify those subjects (dogs) without the LD^34^. However, the relatively crude measures of sensitivity and specificity fail to take into account the cut-off point for a particular test, such as in this case^35^. The area under the ROC curve (AUC) represents the overall accuracy of the diagnostic test. It takes values from 0 to 1, where a value of 0 indicates a perfectly inaccurate test and a value of 1 reflects a perfectly accurate test. In general, an AUC of 0.5 suggests no discrimination (i.e., ability to diagnose patients with and without the disease or condition based on the test), 0.7–0.8 is considered acceptable, 0.8–0.9 is considered excellent, and more than 0.9 is considered outstanding^36^.

The optimal cut-off points were determined by using the Youden index. The Youden index, first introduced to the medical literature by Youden^37^, is a function of sensitivity and specificity and is a commonly used measure of overall diagnostic effectiveness. Sensitivity and specificity for the A1350/A1250 ratio established cut-off points were calculated with their 95% confidence interval.

## Results

**Figure 2:**
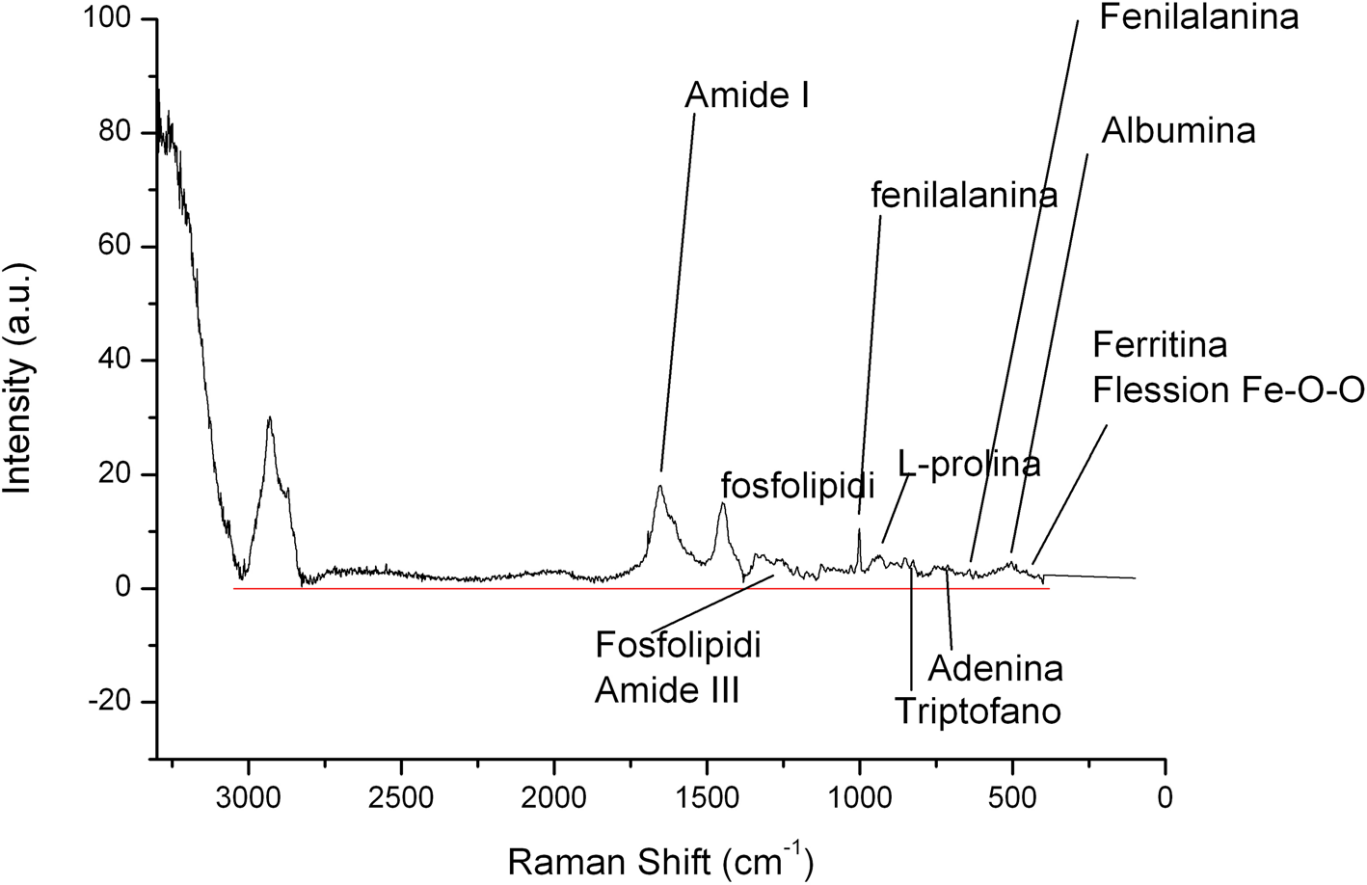
Average Raman spectrum of sera from control group (CG) subjects (n = 11) in the spectral range of 3100–400 cm^−1^, after having performed a baseline correction of each of them in order to compensate eventual technical and/or sample variations, and having normalized them to the total integrated area. The spectrum put in evidence the main typical protein vibrational modes, which derive from the polypeptide backbone (amide bands) and from aromatic and non-aromatic amino acid residue side chains.

**Figure 3:**
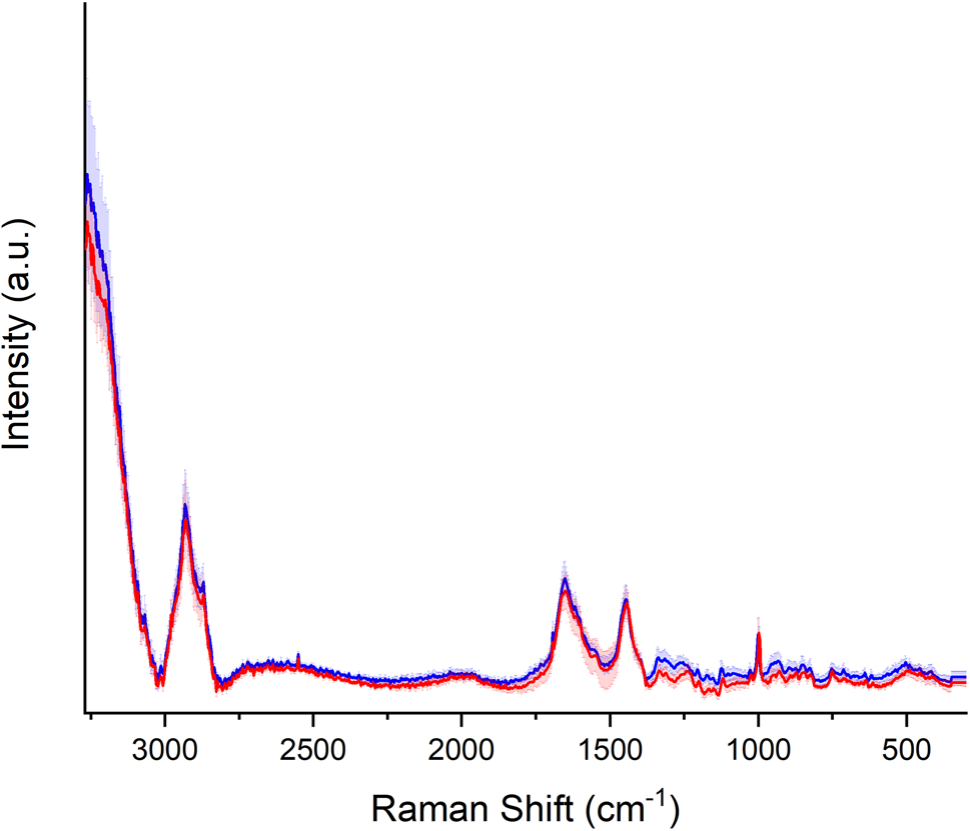
Mean (continuous line) and standard deviation (gradient color) of Raman spectra of LG (red line) and CG (blue line) subjects.

**Figure 4:**
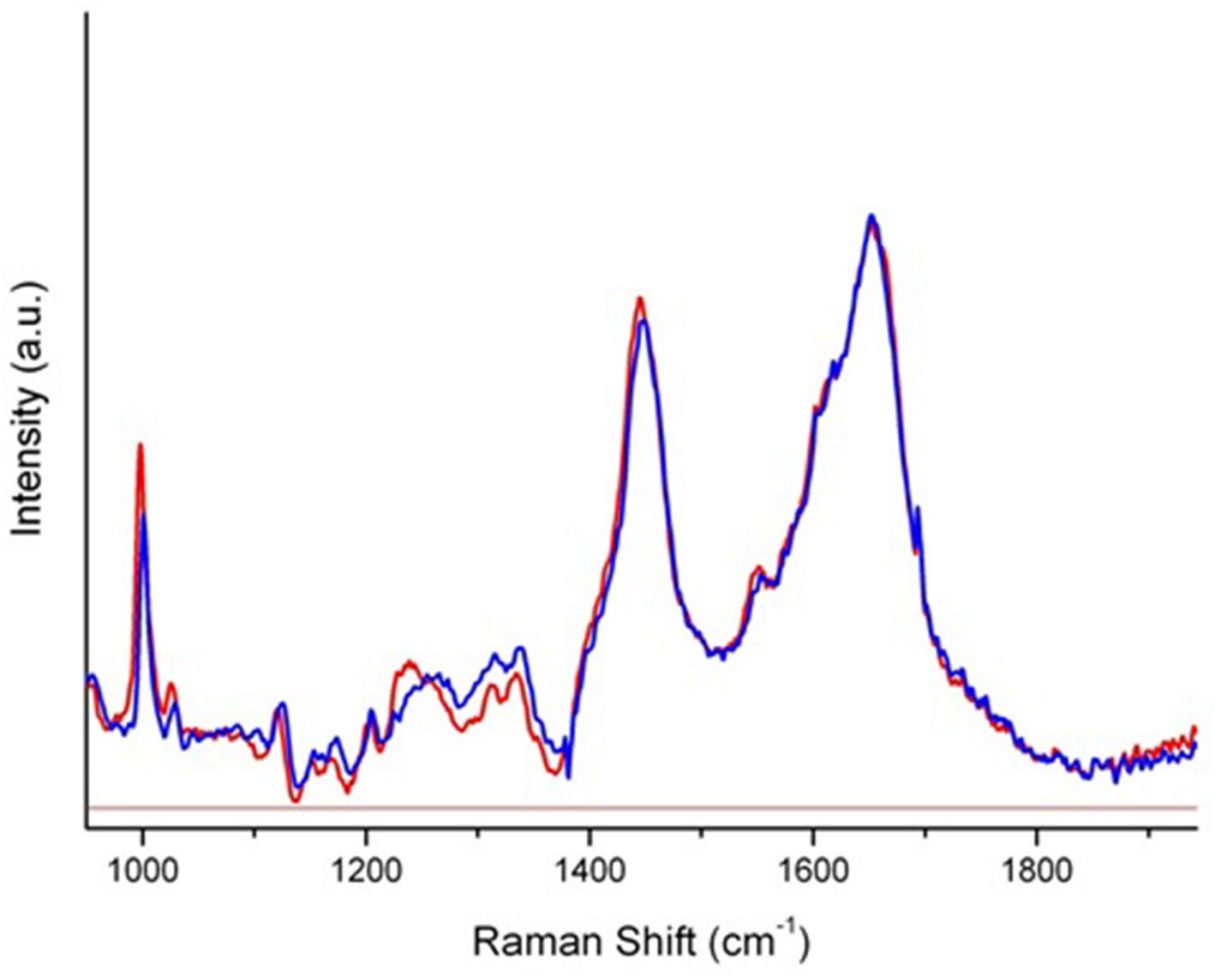
Acquired spectra, obtained from sera of LG (red line) and CG (blue line) subjects, in the region 900–1900 cm^−1^. The attribution of amide III vibration is around the ~ 1300 cm^−1^.

In Fig. 2, we report the average Raman spectrum of sera from CG subjects in the spectral range of 3100–400 cm^−1^, after having performed a baseline correction of each of them in order to compensate eventual technical and/or sample variations. In Fig. 3 we report the mean Raman spectrum and standard deviation (SD) of normalized obtained spectra of LG (red) and CG (blue); in particular, SD is depicted as color gradient. The spectrum shows the main typical protein vibrational modes, which derive from the polypeptide backbone (amide bands) and from aromatic and non-aromatic amino acid residue side chains. The assignment of the main vibrational bands is stated considering the literature and it is reported in Table 2. The acquired spectra, obtained from sera of LG and CG subjects, were visually similar, except in the region 1200–1370 cm^−1^ (Fig. 4), inside it we can distinguish the amide III vibration (~ 1300 cm^−1^). Figure 4 depicts the average Raman spectra of LG (red line) and CG (blue line) subjects in the region 900–1900 cm^−1^.

**Table 2:** Tentative assignment of the main vibrational bands based on the literature.

Center frequency (cm^−1^)	Tentative assignment	References
520	Disulfide band	^28^
759	Ring vibration of tryptophan	^38,39^
830 and 850	Tyrosine doublet	^39^
1000	Phenylalanine	^28,40^
1213–1279 band	Leucine and isoleucine	^29–31^
1280–1370 band	Leucine	^32^
1450 band	CH_2_ scissoring deformation	^41^
1550	Amide II vibration	^28^
1650	Amide I vibration	^42^
2935	C–H stretching vibration	^28,42^

In order to decide the presence of the disease in the involved subjects, for each of them the ratio R of the two areas related to the two bands centered in ~ 1350–1250 cm^−1^ (R = A_1350/A_1250) was computed. Alterations in the protein network as a consequence of the inflammatory state were monitored by evaluating the ratio R and the obtained values for all the analyzed subjects were used for statistical analysis.

**Figure 5:**
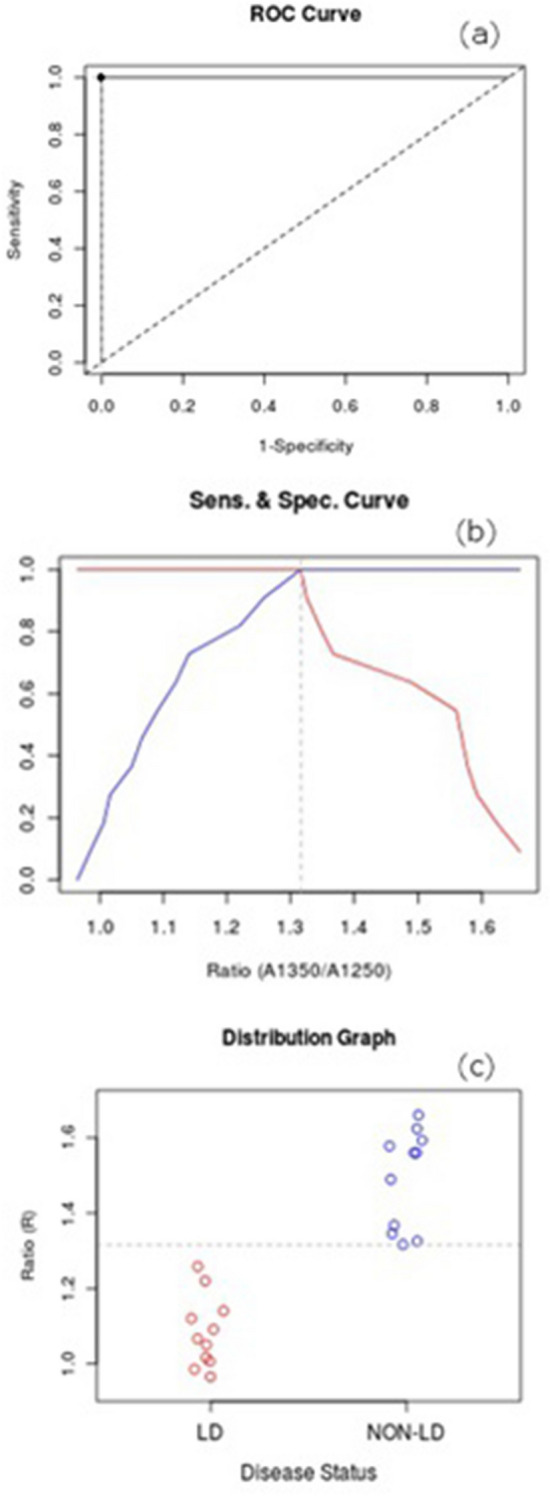
(**a**) ROC curve for the A1350/A1250 ratio (AUC = 1, 95% confidence interval). The diagonal joining the point (0.0) and (1.1) represents the line of equality. The black point corresponds to the 1-Specificity, Sensitivity calculated for the optimal cut-off for correctly identifying Leishmania (LG) or control (CG) subjects. (**b**) Trend of sensitivity (red line) and specificity (blue line) vs. A1350/A1250 ratios. The vertical dot line identifies the optimal cut-off (threshold value = 1.316) and the point of intersection of sensitivity and specificity curves corresponds to the black marker depicted in (**a**). (**c**) Scatter distribution of each group (CG and LG). The optimal cut-off is plotted as the horizontal dot line.

Figure 5a plots the ROC curve for the A1350/A1250 ratio (AUC = 1, 95% confidence interval). In the same figure, the diagonal joining the point (0,0) and (1,1) is also represented (Line of equality) and the black point corresponds to the (1-Specificity, Sensitivity) calculated for the optimal cut-off for correctly identifying LG or CG subjects. Figure 5b shows the trend of sensitivity (red line) and specificity (blue line) vs. A1350/A1250 ratios. The vertical dot line identifies the optimal cut-off (threshold value = 1.316) and the point of intersection of sensitivity and specificity curves corresponds to the black marker depicted in Fig. 5a. Figure 5c displays the scatter distribution of each group (CG and LG). In Fig. 5c, the optimal cut-off is plotted as the horizontal dot line.

## Discussion

This study is a preliminary investigation to verify the applicability of Raman spectroscopy as an alternative methodology for the diagnosis of canine leishmaniasis^27^. Raman Spectroscopy represents a versatile and fast method that allows us to distinguish between healthy and sick animals, also helping during therapeutic monitoring; in fact, Raman Spectroscopy is a useful tool providing data about motions in molecules and in pathological events, it is possible to observe changes, which exert their effects on molecular motions^43^.

The results of the research did not entirely satisfy the hypothesis, although they are very interesting and encouraging, since the spectra obtained in the animals of each group are very homogeneous, practically overlapping them. The clear difference in intensity observed between the two spectra suggests the validity of Raman spectroscopy in the diagnosis of canine leishmaniasis, as recently proposed in studies conducted in mouse models infected with L. braziliensis^44^.

In Fig. 4, it appears clear that, as disease occurs, an evident shift to the shortwave region is observed in spectral frequencies of the band centered at ~ 1250 cm^−1^, corresponding to the region of leucine protein^30^. It is known that a critical point in the host-parasite interaction involves the attachment to and invasion of host macrophages. It occurs through the membrane coating formed by layers of glycolipids such as lipophosphoglycan (LPG), proteophosphoglycan (PPG), and proteins such as MSP/GP63 (major surface protein 63-kDa glycoprotein), PSA-2/GP46 (parasite surface antigen-2 or 46-kDa glycoprotein) and amastin surface proteins, which are expressed by amastigotes during their development^44^. The frequency shift in the region of leucine, observed by Raman spectroscopy in the serum of LG, could be related to the host-parasite interaction. Leucine is a constituent protein of the parasite surface antigen (PSA2) primary structure, which is one of the major antigens of the surface of the L. infantum parasite membrane. The most suggestive functional determinant in the PSA2 primary structure is the presence of leucine rich repeats (LRR). LRRs are primarily known to be involved in protein–protein and protein-glycolipids interactions, whereby the leishmania amastigote interact with host macrophages and resist to complement lysis^45,46^.

Observing the results depicted in Fig. 5, a subject is assessed as affected (positive) if the A1350/A1250 ratio tested marker values in greater than the 1.316 threshold value; otherwise, the subject is diagnosed as NON-LD subject.

Moreover, some authors reported that LRRs are the main epitopes in L. infantum PSA during canine and human visceral leishmaniasis. They showed that the central region of PSA, formed exclusively by LRRs, has a very high antigenic capacity and it is recognized by 100% of the sera from the infected dogs and 40% of the human sera. Thus, the LRRs would be the immunogenic determinants of the PSA during natural leishmania infection, being the responsible of the host antibody response^47^. The modification of the Raman spectrum of amide III and in the leucine region observed in sick subjects could be related to the mechanisms of parasite host interaction. The frequency shift in the region of phospholipids, observed by Raman spectroscopy in the serum of LG, seems also to confirm the key role of lipid metabolism in the pathogenesis of the disease^48–50^. In mammals, the host-Leishmania interaction involves the attachment and invasion of host macrophages, initially by metacyclic promastigotes and subsequently by amastigotes. In particular, it has been shown that the alteration of the pathways of lipid metabolism, the transfer, modification and accumulation of lipids during infection with Leishmania spp. are a key point in disease progression^50^.

Results of the present study showed that Raman spectroscopy could be considered a diagnostic tool for detection of dogs affected by leishmaniasis. Accuracy, sensitivity and specificity of the proposed diagnostic test were determined using Receiver Operating Characteristic (ROC) curve and Youden index (Y), furnishing excellent results, which demonstrated the feasibility of Raman spectroscopy as diagnostic modality for rapid de-tection of LD subjects.

All the results obtained by using Raman Spectroscopy technique were confirmed by those obtained using the methodology reported in CLWG guidelines^26,27^.

The few numbers of animals and its inhomogeinity in breed an age, however, does not allow us to formulate conclusive considerations and further investigations in dog cohorts will be needed, numerically significant, differentiated by clinical status and antibody titer and/or parasitic load (e.g., quantitative PCR).

Another limitation of this study is represented by the presence of only healthy and infected animals with L. infantum. Although the ability to distinguish between healthy and diseased animals is unequivocal, it cannot be excluded that the variation in the Raman spectra may be related to an organic/metabolic perturbation rather than to the specific disease.

## Conclusions

The gold standard for the diagnosis of Leishmania disease in dogs is the consequence of hematological, hematochemical and serological analyses performed during the clinical examination. These exams are resource expansive and time consuming. On the other hand, Raman spectroscopy is a current research topic in the biomedical field that has proved to be easily to perform, and represents a rapid, reproducible and non-invasive technique. It is capable of both providing useful information for understanding the pathogenesis of diseases and expanding their possibility of early diagnosis and evolutionary and therapeutic monitoring. It does not require chemical diagnostic kits; it is non-destructive and the only limit of the analysis is represented by the degradation of the sample itself.

In the present study, a rapid non-destructive and non-invasive diagnostic method for the detection and discrimination of Leishmaniosis is presented. The proposed methodology makes use of Raman spectroscopy in order to investigate the changes in protein structures of dogs’ sera. The results obtained in the present research are to be considered as preliminary, considering the small number of enrolled animals, and the necessity to discern between the Lehismania presence and the metabolic modification due to disease as cause of Raman spectra modifications. Further studies are necessary to consolidate the achievability of Raman spectroscopy as rapid and less expensive diagnosis of Leishmaniasis.

## Data Availability

The datasets used and/or analysed during the current study available from the corresponding author on reasonable request.
